# Investigations Into the Urinary Metabolite Elimination Profile of the Selective Androgen Receptor Modulator S‐23 in Studies Mimicking Contaminated Product Ingestion for Doping Control Purposes

**DOI:** 10.1002/bmc.70090

**Published:** 2025-04-25

**Authors:** Hana Alhalabi, Linus Korsmeier, Andreas Thomas, Mario Thevis

**Affiliations:** ^1^ Center for Preventive Doping Research, Institute of Biochemistry German Sport University Cologne Cologne Germany; ^2^ European Monitoring Center for Emerging Doping Agents (EUMoCEDA) Cologne Germany

**Keywords:** doping, metabolism, S‐23, SARMs, sport

## Abstract

Selective androgen receptor modulators (SARMs) have repeatedly been reason of adverse analytical findings (AAFs) in routine doping controls. Among these, S‐23 has been identified in five AAFs reported in 2022. In addition to intentional doping, inadvertent exposure through contaminated dietary supplements has emerged as a significant concern, purportedly as well as evidently contributing to AAFs involving SARMs. Thus, the differentiation of inadvertent intake and intentional abuse of S‐23 is of growing relevance. This study aimed at investigating the urinary concentration profile of microdosed S‐23 and to characterize the elimination pattern. Single and multidose administration studies with 1, 10, and 50 μg of S‐23 were conducted, and collected urine samples were analyzed by LC–MS/MS following enzymatic hydrolysis and solid‐phase extraction. The analytical method was validated for a semiquantitative detection of S‐23 and characterized by a limit of detection of 1 pg/mL. A total of 18 metabolites was detected in human in vivo samples following oral administration of microdosed S‐23. Moreover, the study demonstrated that a single dose of 1 μg can be detected for an average of up to 253 h, while a single dose of 50 μg can be detected up to 544 h on average.

## 1 Introduction

Anabolic agents are the most frequently detected prohibited drugs in professional sports (WADA 2024a). One class of substances known as SARMs has been showing a growing prevalence since 2014 (I. V. Efimenko et al. 2021; WADA 2024a). Their popularity is further fueled through social media and online marketing including largely unsubstantiated health claims, as indicated by investigations into online research trends concerning SARMs (I. V. Efimenko et al. 2021; Hahamyan et al. 2023; Leciejewska et al. 2024). In vitro experiments have shown that SARMs exhibit agonistic activity on the androgen receptor, primarily in muscle and bone tissues while demonstrating reduced activity within other tissues such as prostate and seminal vesicles (Negro‐Vilar 1999). Therefore, SARMs are widely believed and promoted to be safe in use, especially compared with anabolic androgenic steroids (AAS) (Leciejewska et al. 2024). However, case reports and clinical studies have described several side effects, keeping in mind that the long‐term effects of chronic SARM use are not fully known (Iakov V Efimenko et al. 2022; Machek et al. 2020). The observed side effects comprise serum and lipid level abnormalities, potential increase in the risk of cardiovascular diseases and liver damages as well as potentially reduced testosterone levels, which may negatively impact the hypothalamic–pituitary‐gonadal axis, leading to imbalances in endocrine pathway (Leciejewska et al. 2024), (Machek et al. 2020), (Dalton et al. 2011; FDA 2021; Malave 2023; Mohideen et al. 2023). Despite the substance class undergoing clinical research for several years, no SARM has been approved or commercialized as a pharmaceutical drug, primarily due to the appearing adverse effects, and yet SARMs are available on the black market (Krug et al. 2014; Van Wagoner et al. 2017).

In the context of doping, the relevance of SARMs continues to grow, as indicated by the World Anti‐Doping Agency's (WADA) annual Anti‐Doping Testing Figures Report, showing an increase from 13 adverse analytical findings (AAFs) of SARMs in 2013 (WADA 2015) to 104 AAFs in 2022 (WADA 2024a). Notably, WADA already explicitly prohibited the use of SARMs both in‐and‐out‐of‐competition in 2008 (WADA 2024b). A closer look at SARM S‐23 ([3‐(3‐fluoro‐4‐chlorophenoxy)‐*N*‐(4‐cyano‐3‐trifluoromethylphenyl)‐2‐hydroxy‐2‐methyl‐propionamide]) reveals an recent increase in detection, with one AAF recorded in 2019, zero AAFs in 2020, two in 2021, and five in 2022 (WADA 2021, 2022, 2023a, 2024a).

**FIGURE 1:**
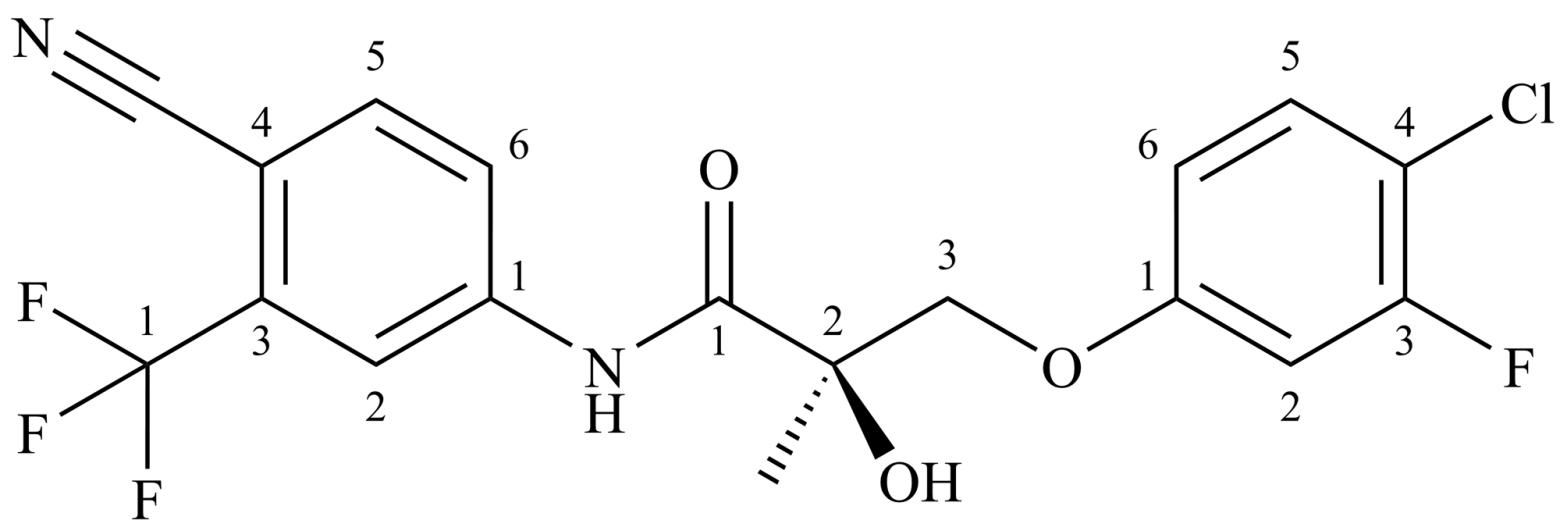
Molecular structure of S‐23.

In animal models, the novel SARM S‐23 (Figure 1) demonstrated a dose‐dependent increase in bone mineral density and lean mass, as well as a reduction in fat mass (Jones et al. 2009). Further, investigations into the potential hormonal role of S‐23 as male contraceptive and as an approach to prevent glucocorticoid‐induced muscle loss were made, although S‐23 never became subject of a clinical trial (Jones et al. 2009; Jones et al. 2010).

In anti‐doping, various scenarios leading to AAFs need to be taken into consideration, including the intended misuse of SARMs as performance enhancing agents as well as unintentional exposure, for example, through transfer or contamination (e.g., of nutritional supplements), representing a serious risk to athletes as part of their respective exposome (USADA 2017). Due to the principle of the strict liability as defined in the World Anti‐Doping Code, athletes might encounter situations of an anti‐doping rule violation (ADRV) whenever a prohibited substance is detected in their doping control specimens (Kornbeck 2016; WADA 2021). Thus, advancing analytical methods that provide insights into elimination profiles is critical, as these data support results management and contribute to the protection of clean athletes.

Hence, within this research project, S‐23 microdose studies were conducted to investigate the human in vivo metabolism and elimination behavior of the drug, simulating contamination scenarios of inadvertent drug intake. These experiments extend previous research, such as the human liver in vitro study and metabolite characterization work that first described a total of 11 phase I and II metabolites as well as an estimation of each metabolite's contribution to the sum of the measured metabolic products (Thevis et al. 2010). In the field of equine anti‐doping research, an in vitro study using horse liver microsomes described a total of eight metabolites (So et al. 2021), while an in vivo study observed S‐23 and seven phase I metabolites in urine (Cutler et al. 2024). The first S‐23 human in vivo study involved a single oral dose of approximately 8 mg, was used to mimic an intended misuse. In this study, besides unconjugated S‐23, a total of two metabolites were identified in urine, while no traces of S‐23 were detected in hair (Ameline et al. 2022).

## 2 Experimental

### 2.1 Reference Compounds, Chemicals, and Reagents

Acetonitrile (ACN), methanol, and ammonium acetate were purchased from Merck (Darmstadt, Germany). The enzyme β‐glucuronidase ( *E. coli* ) was from Roche Diagnostics (Mannheim, Germany). S‐23 reference material was obtained from Hycultec (Beutelsbach, Germany). Ultrapure water was produced with a Barnstead GenPure xCAD Plus from Thermo Scientific (Bremen, Germany). Formic Acid purchased from Carl Roth (Karlsruhe, Germany). Human liver S9 fractions 20 mg/mL (S9 mix) and human liver microsomes (HLM) were obtained from Thermo Scientific (Schwerte, Germany). Nicotinamide adenine dinucleotide phosphate tetrasodium salt (NADPH) and human 3′‐phosphoadenosine‐5′‐phosphosulfate tetralithium salt (≥ 80%) (PAPS) were obtained from Merck (Darmstadt, Germany). Uridine‐5′‐diphosphoglucuronic acid (UDPGA) and D‐saccharic acid‐1,4‐lactone (SL) were from Sigma‐Aldrich (St. Louis, MO, USA). For the preparation of the phosphate buffer (100 mM), 3.54 g sodium dihydrogen phosphate (NaH_2_PO_4_) were dissolved in 250 mL of water (solution A) and 1.75 g of potassium dihydrogen phosphate (KH_2_PO_4_) in 125 mL of water (solution B). Solution B was then added to solution A until a pH of 7.4 was reached (solution C). For the final incubation buffer, 0.276 g MgCl_2_*7 H_2_O were mixed with 250 mL of solution C.

As an internal standard (ISTD), the SARM S‐24 (obtained from an in‐house synthesis according to procedures published elsewhere (Krug et al. 2012; Marhefka et al. 2004; Tucker and Chesterson 1988; Tucker et al. 1988) was used. The molecule structure of S‐24 is closely related to S‐23, only differing in the substitution of the 4‐fluorophenyl B‐ring by a 4‐chloro‐3‐fluorophenyl residue of S‐23. Both SARMs show similar behavior during sample preparation and analysis. As a second ISTD, S‐22 glucuronide was used and obtained from Seibersdorf Laboratories (Seibersdorf, Austria). All standards were stored at −18°C.

The blank urine samples used to conduct the validation were obtained from five healthy female and five healthy male individuals.

### 2.2 In Vitro Metabolism Study

First, 10 μL of a 1 mg/mL S‐23 stock solution was incubated with 20 μL of incubation buffer, gently shaken at 500 rpm for 20 h at 37°C. To this incubation mixture, 10 μL of NADPH, 5 μL of S9 mix, and 5 μL of HLM were added. Subsequently, 10 μL each of UDPGA, SL, PAPS, and NADPH, along with 5 μL of S9 mix and 5 μL of HLM, were introduced to the previously incubated S‐23 mixture. This new solution was incubated again under gentle shaking at 500 rpm for additional 24 h at 37°C.

Control experiments were conducted using substrate‐free (substrate blank) and enzyme‐free (enzyme blank) conditions by adding equivalent volumes, allowing differentiation of metabolites from potential matrix‐associated artifacts. After both incubation steps, 50 μL of 2% acetic acid and 400 μL of ACN were added to the solutions, and proteins were precipitated by centrifugation at 17,000 × *g* for 10 min. The supernatant was transferred to clean Eppendorf protein Lo‐Bind tubes, evaporated under reduced pressure centrifugation and reconstituted in 100 μL of 5 mM ammonium acetate buffer containing 0.1% acetic acid. The final solution was transferred into a HPLC glass vial and an aliquot of 2 μL was injected for analysis.

### 2.3 Human Administration Studies

To investigate the human metabolism and elimination profile of microdosed S‐23 and its metabolites, the following administration studies were conducted with ethical approval granted by the local ethics committee (German Sport University Cologne, 173/2023), and written informed consent.

#### 2.3.1 Single‐Dose Studies (1 × 1, 1 × 10, and 1 × 50 μg of S‐23)

S‐23 was dissolved in ethanol to form a 1 mg/mL stock solution. The stock solution was used to fortify 50 mL drinking yoghurt with single doses of 1, 10 and 50 μg of S‐23. The fortified drinking yoghurt was administered orally to five healthy male volunteers each. Urine samples were collected prior to and for 30 days post administration. Within the first 24 h, every urine sample was collected, followed by urine collection every 4 h on the second day and continuing with once daily in the morning thereafter. The urine volumes from the first 24 h were measured and documented.

#### 2.3.2 Multidose Studies (5 × 1, 5 × 10, and 5 × 50 μg of S‐23)

A 1 mg/mL stock solution of S‐23 in ethanol was used to fortify 50 mL of drinking yogurt with five doses of 1, 10, and 50 μg S‐23 each. The fortified drinking yoghurt was orally administered to five healthy male volunteers on five consecutive days each. Urine samples were collected before administration and for 30 days following the last application. Within the first 24 h, each urine sample was collected; on days 2–5, samples were provided prior to each administration and 2, 4, 8, and 12 h post‐administration. On day 6, samples were collected every 4 h followed by one daily collection in the morning on subsequent days. The potential influence of the yogurt vehicle on the bioavailability of S‐23 was not assessed.

### 2.4 Urine Sample Preparation

First, the specific gravity of all urine samples was determined using a digital refractometer (Kern Optics, Balingen, Germany). Then, two different sample preparation strategies were employed as described in the following:

#### 2.4.1 Enzymatic Hydrolysis Followed by Solid‐Phase Extraction (SPE)

For enzymatic hydrolysis, 2 mL of urine were mixed with 10 μL of S‐24 as ISTD (S‐24 at 10 ng/mL in ACN), 750 μL of phosphate buffer (0.8 M; pH 7) and 50 μL of β‐glucuronidase ( *E. coli* ). Hydrolysis was conducted for 1 h at 50°C using a heating block. The samples were centrifuged at 18,000 × *g* for 5 min to separate precipitated proteins. SPE was performed using CHROMABOND HLB cartridges, 60 μm, 3 mL/60 mg (Macherey‐Nagel, Düren, Germany), pre‐conditioned with 3 mL each of methanol (MeOH) and water. Following sample loading, the cartridges were washed with 3 mL of water, and analytes were eluted with 1 mL of MeOH. The eluate was evaporated in a vacuum centrifuge (2 h at 45°C) and reconstituted in 100 μL of ACN/water (60/40) with 0.1% formic acid. Given the expected high urinary concentrations after multiple administrations of 50 μg S‐23, all samples from the first 6 days post‐administration were reconstituted in 200 μL. The final solution was transferred into a HPLC glass vial and an aliquot of 2 μL was injected for analysis.

#### 2.4.2 SPE Without Enzymatic Hydrolysis

Urine samples (2 mL) were prepared by adding 10 μL S‐22 glucuronide as ISTD (S‐22 glucuronide at 100 ng/mL in ACN) and 500 μL of carbonate buffer (20%, pH 9.5) to adjust the pH at 9.5. SPE was conducted using CHROMABOND HR‐XA, 45 μm, 3 mL/60 mg (Macherey‐Nagel) and cartridges were pre‐conditioned with 3 mL of MeOH and 3 mL of water. After sample loading, cartridges were washed with 3 mL of a 2.5% NH_4_OH aqueous solution, followed by elution with 1 mL of MeOH containing 2% acetic acid. The eluate was evaporated in a vacuum centrifuge (2 h at 45°C) and reconstituted in 100 μL of ACN/water (60/40) with 0.1% formic acid.

### 2.5 Liquid Chromatography‐High Resolution Mass Spectrometry (LC–MS/MS)

LC–MS/MS analyses were performed on a Vanquish UHPLC coupled to a Q Exactive Exploris 480 mass spectrometer (Thermo Fisher, Bremen, Germany). Chromatographic separation was achieved using an EC 4/2 Nucleodur C‐18 Pyramid 3 μm pre‐column and a Poroshell 120 EC‐C18 (4.6 mm × 50 mm, particle size 2.7 μm) analytical column (Agilent, Ratingen, Germany). The temperature of the column chamber was set to 30°C. The mobile phases consisted of 5 mM ammonium acetate buffer with 0.1% acetic acid (solvent A) and ACN (solvent B). The LC gradient, with a total run time of 12.5 min, was set as follows: starting conditions 30% solvent B, 0–1.5 min 50% solvent B, 1.5–7.5 min 86.5% solvent B, then up to 100% solvent B from 7.5 to 9 min. A return to 70% solvent B from 9 to 10.5 min, was followed by re‐equilibration at 30% solvent B for 2 min. The flow rate was maintained at 350 μL/min throughout the entire run.

The mass spectrometer was operated in negative mode with an ionization voltage of −3.5 kV and a transfer capillary temperature of 350°C. Data were acquired by full MS experiments and in product ion scan mode using parallel reaction monitoring (PRM). Full scan mass spectra were recorded within *m/z* 100–650 with a resolution of 45.000 FWHM (at *m/z* 200), while PRM experiments were performed at a resolution of 30.000 FWHM (at *m/z* 200), and a scan range *m/z* 50–650 was recorded. Nitrogen, used as a collision gas and for electrospray ionization, was obtained from a N_2_‐generater (CMC, Eschborn, Germany). The used collision energies were optimized for each analyte and are listed in Table 2. Instrument calibration was performed according to the manufacturers recommendations by using the commercially available standard mixture of caffeine, the tetrapeptide MRFA and Ultramark (Thermo Fischer, Dreieich, Germany).

### 2.6 Data Evaluation

The collected LC–MS/MS data were analyzed using the Trace Finder 5.0 software (Thermo Fisher Scientific). Table 2 provides a summary of product ions for each target analyte, with the most abundant ion highlighted in bold. The detection of target analytes was confirmed based on the presence and matching relative abundance of two precursor/product ion pairs. For the semiquantification of S‐23, the ISTD‐normalized peak areas were calculated from the extracted ion chromatograms of the precursor/product ion pair *m/z* 415 → *m/z* 144. Moreover S‐23 was quantified using an external calibration curve with calibrants at 1, 10, 50, 100, 200, 400, 800, 1000, and 2000 pg/mL. Due to the lack of reference material for the metabolites, qualitative analytical results were obtained and peak area ratios with the ISTD were calculated. All obtained S‐23 concentrations were normalized to a specific gravity of 1.020 according to the following equation (WADA 2018):

$${\text{Conc}}_{\text{corrected}}=\frac{\left(1.020-1\right)}{\mathrm{SG}-1}*{\text{Conc}}_{\text{measured}}$$

### 2.7 Method Validation

The method for the semiquantitative determination of S‐23 following enzymatic hydrolysis and SPE was comprehensively characterized with regard to the following parameters, considering the main parameters of the WADA International Standard for Laboratories 2021 (ISL11) (WADA 2020).

- Selectivity: 10 different blank urine samples obtained from healthy male (*n* = 5) and healthy female (*n* = 5) volunteers were tested for the absence of the target analytes or interfering substances.
- Limit of detection (LOD) and limit of identification (LOI): Six different urine samples were fortified with 0.01, 0.05, 0.1, 1, 5, 10, 25, 100, and 500 pg/mL of S‐23 and analyzed on three consecutive days as described above. The LOD is determined as the concentration where the analyte can be detected in 95% of the samples with one precursor/product ion pair and a S/*N*>3. The LOI is determined as the concentration where the analyte can be identified in 95% of the samples with at least two diagnostic ion transitions in an acceptable range.
- Precision: The precision of the approach was determined at three different S‐23 concentrations (100, 500, and 1000 pg/mL) by analyzing 10 replicates each. The coefficients of variation (CVs) were calculated on the basis of the ISTD‐normalized peak areas.
- Recovery: The recovery was investigated at a S‐23 concentration of 100 pg/mL. 10 blank urine specimens and 10 urine samples fortified with 100 pg/mL of S‐23 were prepared as described above. Prior to LC‐MS/MS analysis, the same concentration of S‐23 was added to the blank extracts and ISTD was added to all urine extracts. Finally, the normalized peak areas of the samples fortified before and after the analyte extraction were compared to estimate the recovery.
- Matrix effects: To investigate ion suppression/enhancement effects, a standard solution of 100 pg/mL of S‐23 and ISTD in a mixture of ACN/water 0.1% formic acid was compared with 10 fortified urine extracts, fortified with the same concentration after sample preparation.
- Correlation: Urine samples were fortified with 1, 10, 50, 100, 200, 400, 800, 1000, and 2000 pg/mL of S‐23 and analyzed on three consecutive days. Calibration curves were generated by using ISTD‐normalized peak areas and correlation was determined by regression analysis.
- Stability: Ten urine samples that were fortified with 100 pg/mL were reanalyzed after 10 days of storage in the instrument autosampler at 10°C.
- Carryover: One sample was fortified with 4000 pg/mL (400% MRPL) and measured followed by three consecutive analysis of three extracted blank samples.

## 3 Results and Discussion

### 3.1 Method Validation

The method employed for a qualitative determination of S‐23 following enzymatic hydrolysis and SPE was comprehensively characterized and the results are listed in Table 1. The approach was found to be highly selective and showed good correlation from 50 to 2000 pg/mL (*R* ^2^ > 0.99) with an estimated LOD of 1 pg/mL. Recovery, matrix effects, stability, carryover and robustness showed acceptable values and were deemed adequate for this application.

**TABLE 1:** Validation results for the qualitative detection of S‐23.

Intraday precision	10.9%	At 100 pg/mL	*n* = 10
Intraday precision	5.8%	At 500 pg/mL	*n* = 10
Intraday precision	7.9%	At 1000 pg/mL	*n* = 10
Interday precision	17.9%	At 100 pg/mL	*n* = 30
Interday precision	6.3%	At 500 pg/mL	*n* = 30
Interday precision	8.9%	At 1000 pg/mL	*n* = 30
LOD	1 pg/mL	0.01–100 pg/mL	*n* = 6
LOI	100 pg/mL	0.01–500 pg/mL	*n* = 6
Selectivity	100%		*n* = 10
Recovery	45.9%–71.6%	At 100 pg/mL	*n* = 10
Matrix effect	103.8%–108.5%	At 100 pg/mL	*n* = 10
Extract stability (after 10 days)	79.3%–100.0%	At 100 pg/mL	*n* = 10
Carryover	0.0%	At 4000 pg/mL	*n* = 1
Correlation (*R* ^2^)	0.9984	50–2000 pg/mL	*n* = 9

### 3.2 Metabolite Identification and Comparison of In Vitro and Human In Vivo Experiments

Following incubation with HLM and liver S9 mix, nine in vitro‐formed metabolites were identified (Table 2). To compare the in vitro‐derived metabolites to those found in urine samples of the human in vivo studies, specimens of the 50 μg single‐dose administration study were investigated. All in vitro*‐*formed metabolites were also detected in vivo, except for metabolite M3. In addition, 10 further metabolites were detected in vivo, yielding a sum of 18 urinary metabolites observed in post‐administration samples (Table 2).

**TABLE 2:** Elemental compositions of deprotonated molecules [M − H]^−^ of S‐23 and M1–M10 with resulting diagnostic product ions using high resolution/high accuracy MS/MS experiments.

Name	Metabolic reaction	[M − H]^−^ (*m/z*)	Elemental comp. (exp)	Error (ppm)	Product ion (*m/z*)	Elemental comp. (exp)	Error (ppm)	RT (min)	ce (%)	In vitro?	In vivo?
S23		415.0478	C_18_H_12_O_3_N_2_ClF_4_	2.6	269.0543	C_12_H_8_O_2_N_2_F_3_	3.35	5.29	30	Yes	Yes
					241.0594	C_11_H_8_ON_2_F_3_	3.32				
					185.0332	C_8_H_4_N_2_F_3_	2.70				
					**144.9861**	C_6_H_3_OClF	3.45				
M1a	Hydroxylation	431.0427	C_18_H_12_O_4_N_2_ClF_4_	3.0	285.0492	C_12_H_8_O_3_N_2_F_3_	2.46	4.35	35	Yes	Yes
					**255.0386**	C_11_H_6_O_2_N_2_F_3_	1.96				
					185.0332	C_8_H_4_N_2_F_3_	1.08				
					144.9861	C_6_H_3_OClF	2.07				
M1b	Hydroxylation	431.0427	C_18_H_12_O_4_N_2_ClF_4_	5.3	269.0543	C_12_H_8_O_2_N_2_F_3_	3.35	3.93	35	Yes	Yes
					185.0332	C_8_H_4_N_2_F_3_	2.70				
					**160.9811**	C_6_H_3_O_2_ClF	3.11				
M1c	Hydroxylation	431.0427	C_18_H_12_O_4_N_2_ClF_4_	2.0	269.0543	C_12_H_8_O_2_N_2_F_3_	3.35	4.21	35	Yes	Yes
					185.0332	C_8_H_4_N_2_F_3_	4.32				
					**160.9811**	C_6_H_3_O_2_ClF	3.11				
M1d	Hydroxylation	431.0427	C_18_H_12_O_4_N_2_ClF_4_	2.5	269.0543	C_12_H_8_O_2_N_2_F_3_	2.97	4.67	35	Yes	Yes
					185.0332	C_8_H_4_N_2_F_3_	2.16				
					**160.9811**	C_6_H_3_O_2_ClF	2.48				
M2a	Bishydroxylation	447.0376	C_18_H_12_O_5_N_2_ClF_4_	—	255.0386	C_11_H_6_O_2_N_2_F_3_	−1.96	3.20	35	No	Yes
					**185.0332**	C_8_H_4_N_2_F_3_	−2.70				
M2b	Bishydroxylation	447.0376	C_18_H_12_O_5_N_2_ClF_4_	—	255.0386	C_11_H_6_O_2_N_2_F_3_	1.96	3.84	35	No	Yes
					285.0492	C_12_H_8_O_3_N_2_F_3_	1.75				
					**160.9811**	C_6_H_3_O_2_ClF	1.86				
M3	O‐dephenylation	287.0649	C_12_H_10_O_3_N_2_F_3_	3.1	257.0543	C_11_H_8_O_2_N_2_F_3_	3.11	2.26	30	Yes	No
					214.0485	C_10_H_7_ONF_3_	2.80				
					213.0281	C_9_H_4_ON_2_F_3_	4.69				
					**185.0332**	C_8_H_4_N_2_F_3_	2.70				
M4	Amide hydrolysis	185.0332	C_8_H_4_N_2_F_3_	−2.1	**158.0223**	C_7_H_3_NF_3_	−1.90	1.25	45	No	Yes
					145.0207	C_8_H_2_N_2_F	−1.38				
					115.0301	C_7_H_3_N_2_	1.74				
M5	Amide hydrolysis and hydroxylation	201.0281	C_8_H_4_ON_2_F_3_	1.4	**181.0218**	C_8_H_3_ON_2_F_2_	2.21	2.62	40	Yes	Yes
					154.0109	C_7_H_2_ONF_2_	1.30				
M6	Glucuronidation	591.0798	C_24_H_20_O_9_N_2_ClF_4_	3.2	445.0864	C_18_H_16_O_8_N_2_F_3_	1.80	6.19	30	Yes	Yes
					415.0478	C_18_H_12_O_3_N_2_ClF_4_	0.96				
					287.0649	C_12_H_10_O_3_N_2_F_3_	2.44				
					269.0543	C_12_H_8_O_2_N_2_F_3_	2.60				
					185.0332	C_8_H_4_N_2_F_3_	2.16				
					**144.9861**	C_6_H_3_OClF	2.76				
M7a	Hydroxylation and glucuronidation	607.0748	C_24_H_20_O_10_N_2_ClF_4_	4.9	431.0427	C_18_H_12_O_4_N_2_ClF_4_	2.32	4.17	30	No	Yes
					337.0131	C_12_H_11_O_8_ClF	4.45				
					255.0386	C_11_H_6_O_2_N_2_F_3_	4.71				
					**160.9811**	C_6_H_3_O_2_ClF	4.35				
M7b	Hydroxylation and glucuronidation	607.0748	C_24_H_20_O_10_N_2_ClF_4_	4.2	431.0427	C_18_H_12_O_4_N_2_ClF_4_	1.39	4.68	30	No	Yes
					269.0543	C_12_H_8_O_2_N_2_F_3_	5.20				
					**160.9811**	C_6_H_3_O_2_ClF	4.97				
M7c	Hydroxylation and glucuronidation	607.0748	C_24_H_20_O_10_N_2_ClF_4_	0.6	431.0427	C_18_H_12_O_4_N_2_ClF_4_	0.46	4.94	30	Yes	Yes
					337.0131	C_12_H_11_O_8_ClF	1.48				
					269.0543	C_12_H_8_O_2_N_2_F_3_	1.12				
					**160.9811**	C_6_H_3_O_2_ClF	1.24				
M7d	Hydroxylation and glucuronidation	607.0748	C_24_H_20_O_10_N_2_ClF_4_	0.8	431.0427	C_18_H_12_O_4_N_2_ClF_4_	−1.39	5.08	30	Yes	Yes
					337.0131	C_12_H_11_O_8_ClF	0.89				
					269.0543	C_12_H_8_O_2_N_2_F_3_	0.37				
					**160.9811**	C_6_H_3_O_2_ClF	1.24				
M8a	Bishydroxylation and glucuronidation	623.0697	C_24_H_20_O_11_N_2_ClF_4_	—	447.0376	C_18_H_12_O_5_N_2_ClF_4_	9.84	4.17	30	No	Yes
					337.0131	C_12_H_11_O_8_ClF	4.15				
					285.0492	C_12_H_8_O_3_N_2_F_3_	8.42				
					255.0386	C_11_H_6_O_2_N_2_F_3_	3.92				
					**160.9811**	C_6_H_3_O_2_ClF	3.73				
M8b	Bishydroxylation and glucuronidation	623.0697	C_24_H_20_O_11_N_2_ClF_4_	—	447.0376	C_18_H_12_O_5_N_2_ClF_4_	5.14	4.32	30	No	Yes
					337.0131	C_12_H_11_O_8_ClF	7.42				
					285.0492	C_12_H_8_O_3_N_2_F_3_	9.82				
					255.0386	C_11_H_6_O_2_N_2_F_3_	4.31				
					**160.9811**	C_6_H_3_O_2_ClF	5.59				
M8c	Bishydroxylation and glucuronidation	623.0697	C_24_H_20_O_11_N_2_ClF_4_	—	447.0376	C_18_H_12_O_5_N_2_ClF_4_	8.51	3.87	30	No	Yes
					**269.0543**	C_12_H_8_O_2_N_2_F_3_	4.09				
					176.9760	C_6_H_3_O_3_ClF	3.39				
M9	O‐dephenylation and glucuronidation	463.0969	C_18_H_18_F_3_N_2_O_9_	6.91	287.0649	C_12_H_10_O_3_N_2_F_3_	0.35	2.29	35	No	Yes
					257.0543	C_11_H_8_O_2_N_2_F_3_	1.95				
					241.0594	C_11_H_8_ON_2_F_3_	2.07				
					**185.0332**	C_8_H_4_N_2_F_3_	3.24				
M10	Amide hydrolysis, hydroxylation, and sulfation	280.9849	C_8_H_4_O_4_N_2_F_3_S	4.63	**201.0281**	C_8_H_4_ON_2_F_3_	1.99	1.33	40	No	Yes
					181.0218	C_8_H_3_ON_2_F_2_	3.31				

*Note:* Product ions used to calculate peak/ISTD ratios are expressed in bold.

The formation of M1a is suggested to result from the hydroxylation of the methyl group within the propionamide core in analogy to the structure previously proposed for S‐22 (Walpurgis et al. 2020). The postulated structure for M1a, as depicted in Figure 3b, is supported by the presence of product ions indicative for an intact B‐ring at *m/z* 144 as well as *m/z* 285, and the absence of the ion at *m/z* 269. The elimination of formaldehyde (−30 Da) from *m/z* 285 can then furnish the base peak at *m/z* 255 (Thevis et al. 2010). Alternatively, the hydroxylation leading to M1a could be located at the propionamide chain's position 3 as suggested by Taoussi et al. (2024) for S‐22. For the metabolites M1b/c/d, hydroxylations are suggested to occur at the aromatic B‐ring carbon atoms as evidenced by the presence of the product ion at *m/z* 160, as well as the absence of *m/z* 285 and *m/z* 144 (e.g., Figure 3b,c).

Four hydroxy‐glucuronide metabolites, M7 a/b/c/d, were observed, though only three hydroxylation sites are available on the B‐ring. M7 a/c/d produce product ions at *m/z* 337 (e.g., Figure 3d,e), indicating glucuronidation at the aromatic hydroxyl group. Conversely, the MS/MS spectrum of M7b lacks an ion at *m/z* 337 (Table 2), suggesting that the glucuronidation occurs at the hydroxyl group on the chiral carbon. Since the chromatographic retention times of M7a and the bishydroxylated metabolite (M8b, *vide infra*)) are very similar and an in‐source dissociation (and thereby H_2_O elimination) of M8b cannot be excluded, the product ion mass spectrum attributed to M7a might be affected by or even result from the M8b species (Table 2). A glucuronidated derivative of metabolite M1a was not detected.

**FIGURE 2:**
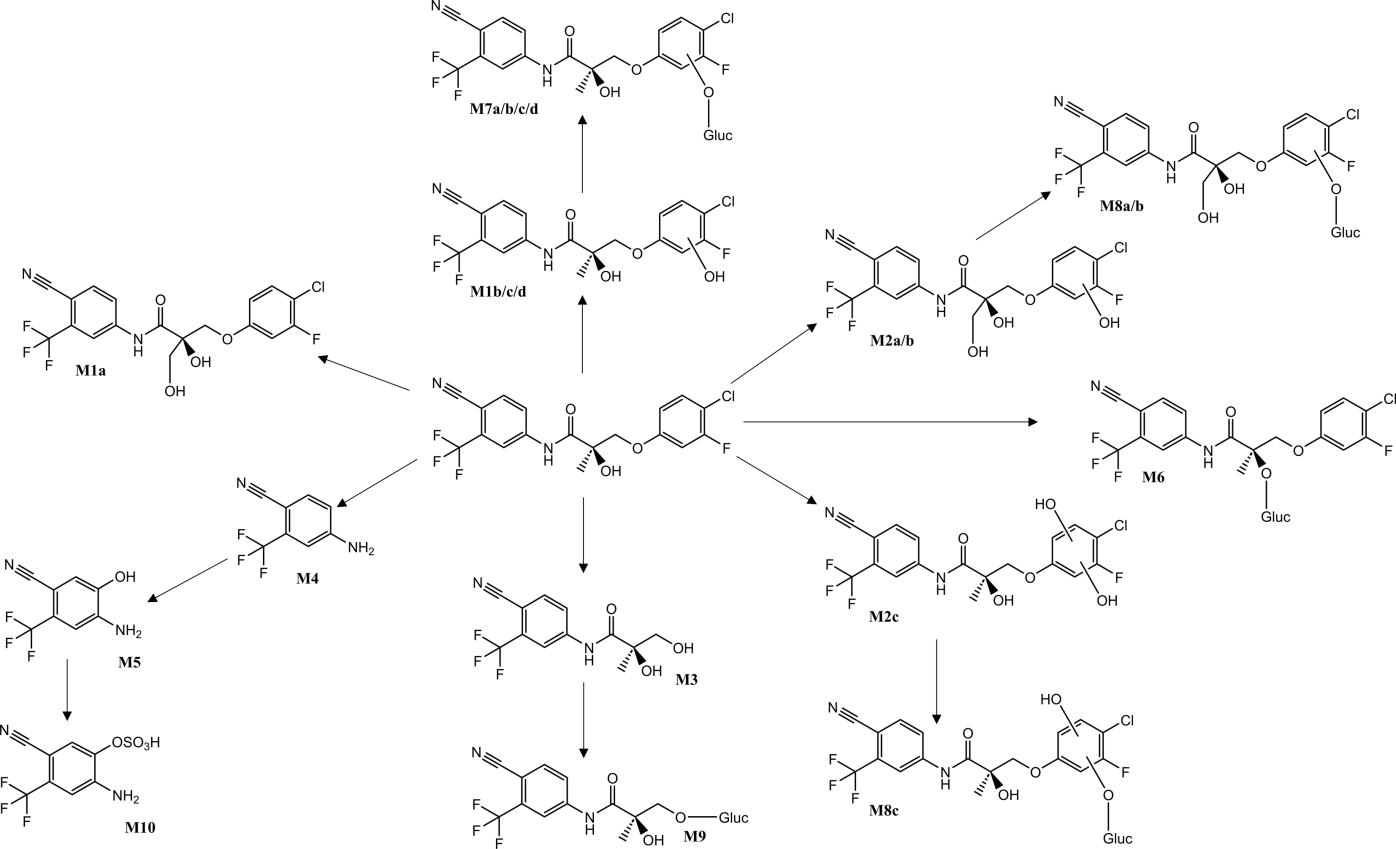
Structures of S‐23 and tentatively identified metabolites in vitro and in vivo*.*

**FIGURE 3:**
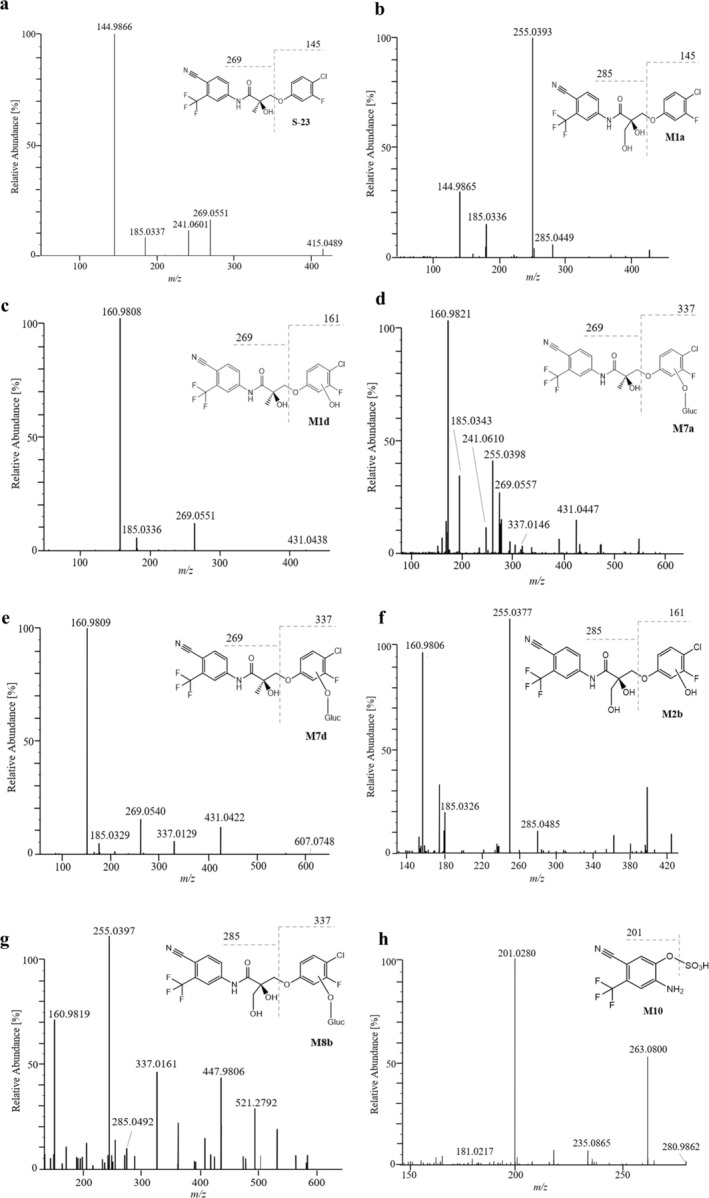
Product ion mass spectra of human in vivo‐derived metabolites of S‐23.

Two bishydroxylated metabolites M2a and M2b (Figure 3f), suggested to exhibit both aliphatic and aromatic hydroxylation, were identified as novel human in vivo metabolites and were not observed in in vitro experiments. These metabolites underwent further glucuronidation, producing M8a and M8b, with glucuronidation specifically occurring at the aromatic hydroxyl group (Figure 2). This was supported by the detection of the ion at *m/z* 337, corresponding to the B‐ring conjugated with glucuronic acid (Figure 3g). However, as for metabolite M2c only a single diagnostic ion was detected, it was excluded from further characterization experiments and, thus, also from Table 2.

The metabolite M9, proposed to be formed via O‐dephenylation and glucuronidation, represents another in vivo metabolite, not detected in vitro. The unconjugated species M3 was not detected in vivo, as mentioned above. The metabolites M4, M5, and M10 were previously described by So et al. (2021) after an in vitro experiment using horse liver microsomes, and all three were also detected in vivo at variable abundance (Figure 3h).

Following in vitro and in vivo phase I metabolism, M1d is the most abundantly formed metabolite. In vitro, the second most abundant metabolite is M3, which is not detected in vivo. Other metabolites formed in relevant amounts in vitro, in descending order of abundance, include M1b, M5, M1a, and M1c. In vivo, the metabolites formed, ranked by abundance, are M1b, M1c, M2b, M1a, and M10. Regarding phase II metabolism, M6 is the predominant metabolite formed in vitro, which aligns with the results reported by Thevis et al. (2010). The second most abundant metabolite is M7d, which is also observed in vivo.

Given that S‐23 contains a chiral carbon atom, enzymatic racemization at this center may be conceivable, as has been observed among pharmaceutical drugs (Nguyen et al. 2006). In order to probe for potential rearrangement reactions, a urine sample collected following the intake of 50 μg of S‐23 was analyzed using an assay that was shown to allow for the separation of enantiomers of S‐22 (Krug and Thevis 2024). The obtained results indicated that only a single enantiomer of S‐23 was excreted, suggesting that racemization or chemical inversion of S‐23 is unlikely to occur.

### 3.3 Single‐Dose Administration Studies and Elimination Profiles

To support a qualitative evaluation of excreted S‐23 and its metabolites, all urine samples were subjected to enzymatic hydrolysis for the detection of unconjugated phase I metabolites (work up method a). Following a single‐dose administration of 1, 10, and 50 μg of S‐23, the intact unconjugated compound, along with three mono‐hydroxylated metabolites M1b/c/d, as well as M2b and M10, were identified (Figure 2). Intact S‐23 and the metabolite M1d emerged as the major urinary metabolites. Analysis using work‐up method b, which excluded enzymatic hydrolysis, revealed that both S‐23 and M1d are primarily excreted as glucuronide conjugates, identified as M6 and M7d, respectively. Notably, M1c and M10 were detected following an intake of 10 and 50 μg, while M2b was detected after the intake of 50 μg. While M1b was consistently detected across all doses, M1d remained the most prevalent metabolite. These findings are consistent with a previous human in vivo study by Ameline et al. (2022).

**FIGURE 4:**
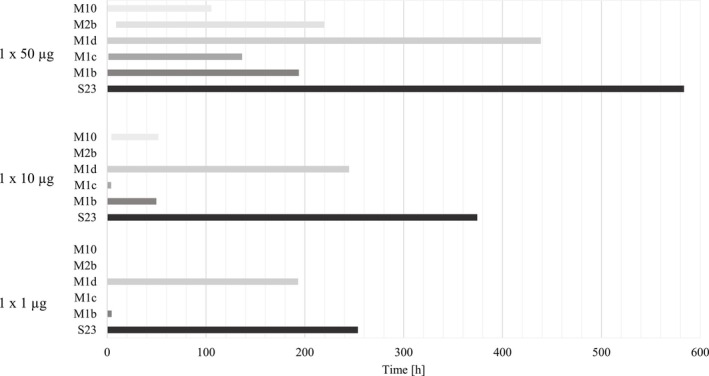
Detection windows of S‐23 and its metabolites after ingestion of one dosage of 50, 10, and 1 μg of S‐23 following enzymatic hydrolysis (work up method a).

Intact S‐23 was detected in urine for 544 h following a single oral 50 μg dose, with an average peak concentration of 1790 pg/mL reached after 18.5 h (Table 3). For comparison, S‐22 was detectable for 144 to 264 h after administration of a single dose of 50 μg, highlighting the extend of the detectability of S‐23 (Walpurgis et al. 2020). The metabolite M1d was detectable for an average time period of 438 h, offering no extension of the detection window. M1b was only detectable for 195 h after administration (Figure 4). Due to the lack of reference material for the metabolic products, absolute quantification was not accomplished. The highest peak area for M1d was observed after 10 h and for M1b after 11 h (Figure 4). Minor metabolites M10 and M2b were detectable up to 105 and 219 h, though M2b was observed in three out of five volunteers (Figure 4).

**TABLE 3:** Summary of detection window and concentration details for S‐23 after single and multidose administration.

Dosage	t_max_ (h)	c_max_ (ng/mL)	Max. detection window (h)
1 × 1 μg	4–12	53–144	213–360
1 × 10 μg	4–12	192–553	235–430
1 × 50 μg	4–22	1185–2792	429–544
5 × 1 μg	80–116	87–468	214–384
5 × 10 μg	80–100	660–1966	309–433
5 × 50 μg	93–100	2692–14,983	500–568

Following the administration of a 10 μg dose, intact S‐23 was detected for 357 h, with an average peak concentration of 387 pg/mL at 11 h (Table 3). M1d was observed for 244 h, peaking at 3 h, while the M1b was detectable for 49 h. The minor metabolite M10 was present between 4 and 51 h post‐administration, and trace amounts of M1c were found in one volunteer's urine up to 4 h post‐application. After the intake of 1 μg, the maximum detection window for intact S‐23 was 193 h, with an average peak concentration of 96 pg/mL at 7 h (Table 3). M1d was detectable for 193 h, while M1b was only detectable for 4 h and could only be observed in one out of five volunteers. No additional metabolites were identified after intake of 1 μg S‐23 (Figure 4).

Detailed examination of the elimination profiles for S‐23 across all three applied doses showed that higher doses resulted in slightly later peak maxima and increased peak concentrations, extending the detection window (Figure 6). Noteworthy is that these prolonged detection times are attributed to the low limit of detection (LOD) of 1 pg/mL, while always meeting the criteria for a secondary diagnostic ion and adhering to WADA criteria for mass spectrometric analyte identification (WADA 2023b). Additionally, evaluations of the excretion rates of S‐23 revealed that 5%–10% of S‐23 was excreted within 24 h across all doses, with no observable dose‐dependency trend.

Both the intact drug and all metabolites exhibited similar or identical excretion profiles, differing primarily in peak signal intensity. All excretion profiles display a secondary, later‐occurring and less intense peak, consistent with the biphasic excretion observed by Ameline et al. (2022). Moreover, higher doses appear to delay the onset of the secondary peak. The biphasic excretion profile reported for microdosed S‐22 similarly reflects this pattern (Walpurgis et al. 2020). This could be explained by a high protein binding behavior, similar to that observed for the pharmaceutical bicalutamide, which shares a comparable molecular core structure (Cockshott 2004).

### 3.4 Multidose Administration Studies and Elimination Profiles

The multiple‐dose administration aimed at simulating a consecutive daily intake, providing insights into the elimination behavior of S‐23 under such scenarios of regular intake, representing a common usage of dietary supplements. The data presented here were obtained using the sample preparation method a, which included enzymatic hydrolysis. Following five consecutively administered doses of 1, 10, and 50 μg of S‐23, distinct interindividual variability in excretion was observed over the first 5 days, with later urine samples showing reduced variability.

**FIGURE 5:**
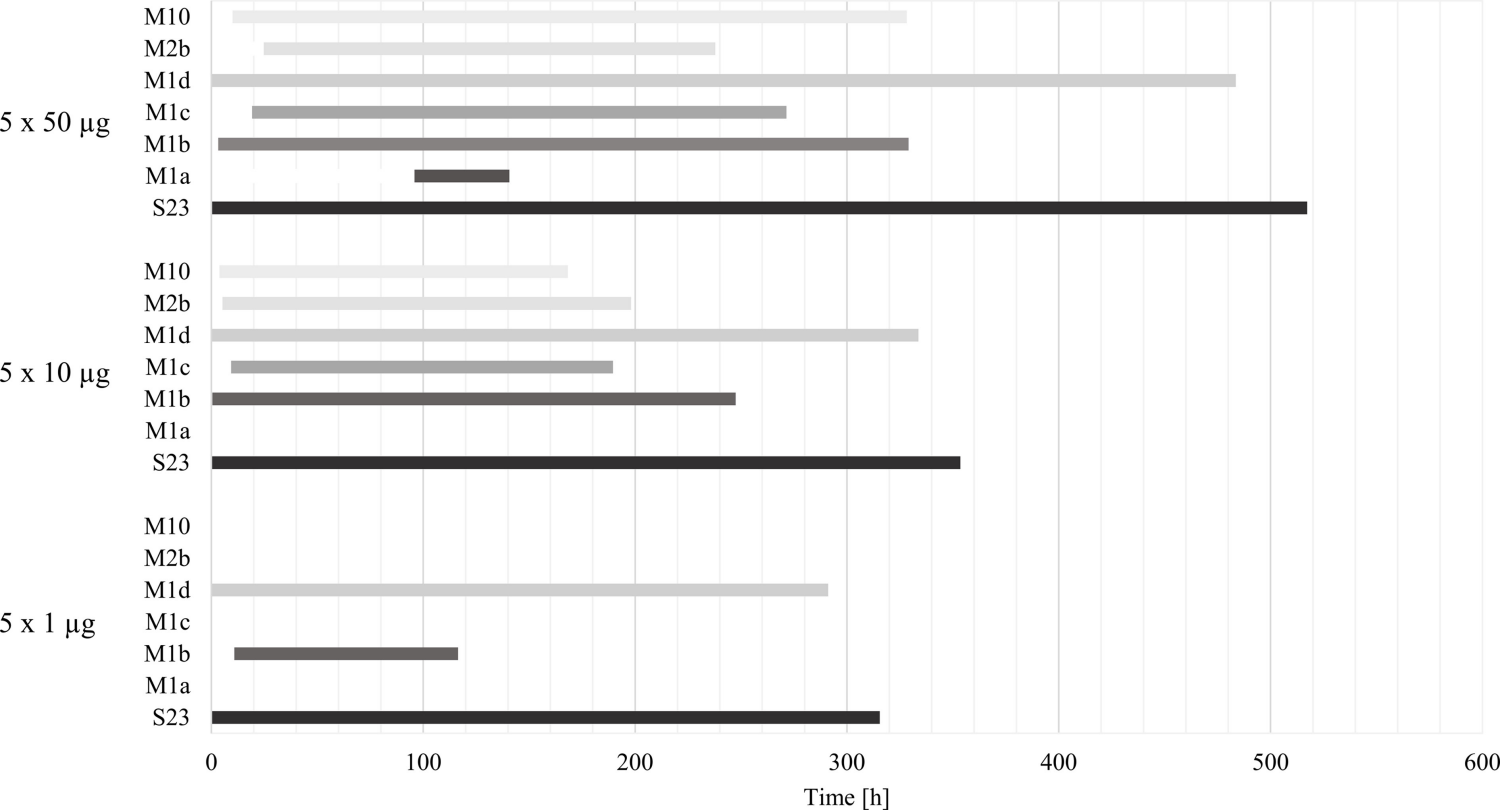
Detection windows of S‐23 and its metabolites after ingestion of five dosages of 50, 10 and 1 μg of S‐23 following enzymatic hydrolysis (work up method a).

Along with the intact unconjugated compound, four mono‐hydroxylated metabolites M1a/b/c/d, as well as M2b and M10, were identified (Figure 2). Consistent with the single‐dose study, intact unconjugated S‐23 and the mono‐hydroxylated metabolite M1d, were found to be the major urinary metabolites, following enzymatic hydrolysis. For the multiple administration of 50 μg of S‐23, the minor metabolite M1a was detectable in samples of four out of five volunteers. Minor metabolites M1c, M2b, and M10 were detectable after 10 and 50 μg doses (Figure 5), while the more prevalent M1b was detectable after each dosage, aligning with the results of the single dose study.

Following 5 × 50 μg S‐23 doses, intact S‐23 was detected for an average of 568 h, with a peak concentration of 6331 pg/mL reached 101 h after the last dose (Table 3). The major metabolite M1d was detectable for an average of 483 h, not extending the detection window further compared with the parent compound, with maximum signal areas detected at 76 h (Figure 5). M1b was detectable for 329 h post first administration, while the minor metabolite M1a was detectable between 95 and 140 h and was thus first detected after 4 to 5 consecutive administrations. M2b and M10 were detectable from 24 to 237 h and 10 to 328 h post first application, respectively (Figure 5). Following a 5 × 10 μg dose, intact S‐23 was detected for an average of 353 h, reaching a peak concentration of 1454 pg/mL at 88 h (Table 3). M1d was detectable for 333 h, peaking at 84 h, while M1b was observed for 247 h. Minor metabolites M1c, M2b, and M10 were detected from 9 to 189 h, 5 to 198 h, and 3 to 168 h, respectively. Regarding the 5 × 1 μg dose study, intact S‐23 showed a maximum detection window of 315 h on average, with a peak concentration of 241 pg/mL at 100 h (Table 3). M1d was detectable for 291 h, while M1b was only detectable between 10 and 116 h and was observed in only one out of five volunteers. The metabolites M1a, M1c, M2b, and M10 were not identified following five 1 μg dosages of S‐23 (Figure 5).

**FIGURE 6:**
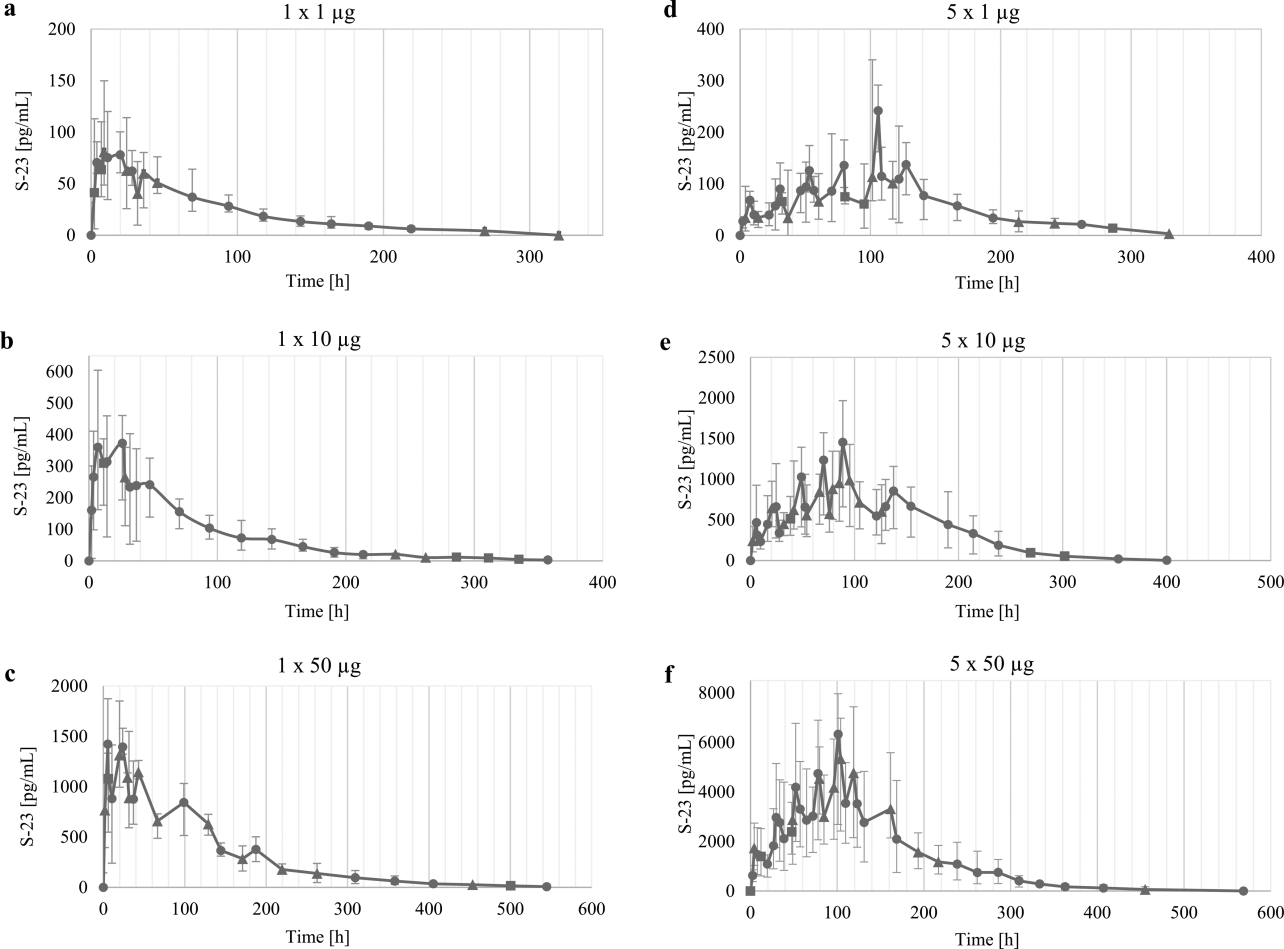
Elimination profiles after single (left) and multiple (right) doses of 1, 10, and 50 μg of S‐23 following enzymatic hydrolysis (work up method a). Shown are averages of multiple data points with error bars to indicate maximum and minimum values. The number of underlying data points shown as circle (5), triangle (4), and square (3).

Detailed investigation of the excretion profiles in the multiple‐dose study (Figure 6) revealed that each peak following consecutive ingestion was higher than the previous, suggesting the accumulation of S‐23 under the chosen conditions. After the final dose, a later and smaller secondary peak was observed, consistent with the biphasic excretion pattern noted in the single‐dose study.

### 3.5 Metabolic Profile

Plotting the natural logarithm of the measured S‐23 concentrations from the single‐dose studies against time produces a straight line with a negative slope, indicating first‐order elimination kinetics. It is generally not feasible to extrapolate the initial concentration or time of ingestion from a single urine measurement for compounds that exhibit first‐order elimination, as the elimination rate is directly proportional to the concentration, which continuously decreases as the drug undergoes metabolism and excretion (UniversityofLausanne 2024; Yartsev 2015). For that reason, investigations of systematic shifts of metabolite ratios over time were performed, to estimate the potential time of ingestion, as Wagener et al. (2022) demonstrated for the SARM LGD‐4033. As mentioned above, the intact drug and all metabolites exhibited similar or identical excretion profiles, indicating that none of the metabolite ratios are suitable for reliable back‐calculation to the time of ingestion. Nevertheless, the results obtained from this study can be utilized to assess the plausibility of an inadvertent intake through contaminated dietary supplements, when comparing the measured S‐23 concentration and the presence or absence of metabolites.

Following the application of work‐up method a, which included enzymatic hydrolysis, the mono‐hydroxylated metabolite M1d, along with unconjugated S‐23, emerged as a primary urinary metabolite, strengthening the specificity of S‐23 detection. However, M1d did not extend the detection window, with unconjugated S‐23 remaining the longest‐detectable analyte. Detection of only unconjugated S‐23 without any metabolites alongside, would suggest an ingestion that took place longer in the past, rather than a recent, regular use, typical for the intake of dietary supplements.

Short‐lived metabolites such as M1c, M2b, and M10 show potential as indicators for recent ingestion. Metabolite M1a, only detected after the fourth or fifth consecutive dosage of 50 μg of S‐23, indicates a regular ingestion, typical for dietary supplements use. Moreover, M1a remains detectable for roughly 45 h, suggesting a very recent intake. These finding could be valuable in cases where an unintended intake through contaminated dietary supplements is suspected, particularly if the ingestion occurred recently.

A previous human in vivo study by Ameline et al. (2022) reported the detection of a monohydroxylated and a bishydroxylated metabolite, following the administration of approximately 8 mg of S‐23. Both metabolites were also detected after microdosed S‐23, which thus do not aid in differentiating doping and inadvertent exposure scenarios. Nevertheless, Ameline et al. (2022) found the monohydroxylated metabolite to be the major metabolite, aligning with the findings of this study.

### 3.6 Application to Routine Doping Controls

In a routine doping control sample, a urinary concentration of 670 pg/mL of S‐23 was detected. In addition to unconjugated S‐23, the major metabolite M1d was detected, as well as M1b. Minor metabolites M1c, M2b, and M10 were also detectable, while M1a was not observed.

Further, a signal attributed to the metabolite M2c, which could not be definitively identified in elimination study samples of this project, was observed using this sample. Metabolite M2a, which was described for human in vivo metabolism but did not show relevance for the evaluation of the administration study conducted here, was also detectable but exhibited only minor signal abundances.

The measured S‐23 concentration in connection with the detected minor metabolites, are compatible with the athlete having been exposed to low (10–50 μg) amounts of the drug in temporal proximity to the event of the doping control sample collection. In consideration of the existence of S‐23‐contaminated dietary supplements as confirmed in the context of follow‐up investigations into AAFs with S‐23, the scenario of an inadvertent intake of the drug cannot be excluded.

## 4 Conclusion

Recent advancements in analytical sensitivity significantly enhance routine doping controls, enabling the prolonged detection of previously undetectable illicit drug administrations. However, the increased sensitivity highlights the need to distinguish between deliberate doping and unintentional exposure to prohibited substances, as the risk of an unintended AAFs for athletes also increases. This human microdose elimination study aimed to provide critical data for decision‐making processes in anti‐doping regarding the SARM S‐23.

The urinary elimination patterns of metabolites and the concentration profiles of S‐23 following oral administration were systematically investigated by performing controlled elimination studies. The collected urine samples were analyzed using the developed LC–MS/MS method, which was validated for the qualitative detection of unconjugated S‐23 in accordance with WADA criteria. In addition to S‐23, 18 metabolites were detected in urine after oral administration of microdosed S‐23. The aliphatic mono‐hydroxylated metabolite M1a was previously described for S‐22 and an analogous biotransformation product was identified also for S‐23. Further, the bis‐hydroxylated metabolites M2b/c and the O‐dephenylated and glucuronidated M9 metabolite were identified as new additions to the metabolic scheme, and all other metabolites were in accordance with previous publications (So et al. 2021; Thevis et al. 2010).

The study design, which included once‐off as well as consecutive administrations of S‐23 mimicking the singular but also repeated exposure reflecting, for example, the intake of contaminated dietary supplements, provides first data on urinary detection windows and elimination patterns for S‐23 and its metabolites under such circumstances. A systematic shift of metabolite ratios over time estimating the time of drug ingestion, was not observed. Nevertheless, urinary metabolite elimination profiles such as those presented in this study can be applied to situations where assessing the plausibility of a scenario of drug exposure is required, and the described detection windows provide a general understanding of how long a presumed contamination remains detectable and which metabolites are more likely to coexist.

## Author Contributions

H. Alhalabi conducted elimination studies; sample preparation and analysis work; performed result interpretation; and wrote the original draft. H. Alhalabi, L. Korsmeier, A. Thomas, and M. Thevis planned and designed the project, and reviewed and edited the final manuscript.

## Ethics Statement

Ethical approval was granted by the ethics committee of the German Sport University Cologne (Nr.173/2023). All volunteers gave their informed consent for participation and publication in anonymized form.

## Conflicts of Interest

The authors declare no conflicts of interest.

## Data Availability

The datasets generated and analyzed as part of this study are available upon request from the corresponding author.
